# Intraoperative Disinfection by Pulse Irrigation with Povidone-Iodine Solution in Spine Surgery

**DOI:** 10.1155/2017/7218918

**Published:** 2017-10-02

**Authors:** Vincenzo De Luna, Federico Mancini, Fernando De Maio, Gabriele Bernardi, Ernesto Ippolito, Roberto Caterini

**Affiliations:** Department of Orthopaedic Surgery, University of Rome Tor Vergata, Viale Oxford 81, 00133 Rome, Italy

## Abstract

**Background:**

Deep wound infection in spine surgery is a debilitating complication for patients and increases costs. The objective of this prospective study was to evaluate the efficacy of wound pulse irrigation with a dilute povidone-iodine solution in the prevention of surgical site infection.

**Methods:**

50 patients undergoing spinal surgery were randomly divided into two groups (A and B) of 25 patients each. In group A, wounds were irrigated with dilute (3%) povidone-iodine solution through a low-pressure pulsatile device. In group B, wounds were irrigated with saline solution through a bulb syringe. In both groups, specimens for bacterial culture were harvested from surgical site before and after irrigation.

**Results:**

In group A, no surgical site infection occurred; in group B, deep wound infection was observed in 3 patients. In both groups, before irrigation some cultures have been found positive for bacterial contamination.

**Conclusion:**

Our study seems to support the idea that low-pressure pulsating lavage of surgical wounds with povidone-iodine diluted to a nontoxic concentration of 3% is an effective therapeutic adjunct measure to prevent surgical site infection in spine surgery. However, the number of the enrolled patients is small and a significant statistical analysis is not practicable. This trial is registered with NCT03249363.

## 1. Introduction

Surgical site infection (SSI) in spine surgery is a serious complication in terms of healthy status of the patient, clinical outcome, and cost for the community. SSI in spine surgery usually requires prolonged antibiotic therapy and one or more surgical debridement operations that can aggravate patient morbidity. The causes of SSI are multifactorial, and they comprise patient and procedure specific risks. According to the best evidenced studies, well summarized by Schuster et al. [1], age (>60 years), diabetes, malnutrition, obesity, ASA score ≥ 3, higher glucose level, transfusions, posterior approach, and duration of surgery are the preoperative and intraoperative risk factors in spine surgery for which a statistically significant association with SSI has been reported. In 1991, the National Nosocomial Infections Surveillance System (NNIS) introduced Infection Risk Index (IRI) to monitor trends in infections and risk factors [2]. It permitted comparing the infection rates, considering the confounding factors secondary to the different types of surgery, to the risk of endogenous contamination and to the general clinical aspect of the patient. It was developed to predict a surgical patient's risk of acquiring a surgical wound infection with an index score ranging from 0 to 3 (Table 1).

Use of perioperative antibiotic treatment has been well supported by retrospective studies and randomized trials, and it should be utilized in any patients undergoing spine surgery [3–6]. However, despite antibiotic prophylaxis, studies on adult and on children report an overall infection rate ranging from 0.4% to 20% after spinal surgery [7–11]. In addition to antibiotic prophylaxis, many perioperative adjuncts have been used to reduce infection rates in spine surgery but few studies, with a low level of evidence, have been published on these measures [12–20]. The most utilized adjunct measures for preventing postoperative SSI are wound irrigation with povidone-iodine (PVP-I) or hydrogen peroxide and saline solution, silver impregnated dressing, closed wound suction drainage, and use of an ultraclean air technology in the operating room. With regard to these measures in spinal surgery, the literature has shown a “moderate,” “low,” or “very low” level of evidence to support their efficacy in reducing the infection rate [13, 14]. PVP-I is often used as a surgical scrub and for antiseptic prophylaxis in open wounds and treatment of superficial and deep infections. The antiseptic function of PVP-I is characterized by a complex of polyvinylpyrrilidine and triiodine ions that acts against cell walls and inhibits the release of pathogenic factors, even of highly resistant microbiological organism [15, 16]. In a dilution of 1 : 25 to 1 : 200 (0.5–4% Poviderm), it has been considered, compared to other irrigating fluids such as soap, antibiotics, and chlorhexidine, the one with the greatest bactericidal efficacy and the lowest cell toxicity [21–23]. Often in orthopaedic surgery, PVP-I solution is utilized through a pulsatile irrigation device which combines irrigation lavage or pulsatile debridement technique with rapid suction removal of effluent. Many authors consider pulsed lavage more effective than bulb syringe in wound irrigation [24–26].

The purpose of this study was to evaluate the efficacy, in preventing SSI, of intraoperative pulsatile irrigation with a 2000 ml saline solution of PVP-iodine in a group of patients undergoing complex spine surgery with a posterior approach. To confirm and better assess the efficacy of intraoperative irrigation, specimens for bacterial culture were harvested by swabs from muscular tissue before and after irrigation of the wounds.

## 2. Materials and Methods

This is a single-center prospective cohort study of a total of 50 consecutive patients (20 males, 30 females; mean age of 41.7 years), operated from 2010 to 2012 for scoliosis. None of the patients showed clinical signs of infection before surgery, and for all of them it was the primary spinal surgery procedure. Operative treatments included 50 instrumented arthrodeses for scoliosis (21 degenerative, 27 idiopathic, and 2 congenital). All the surgeries were performed through posterior central surgical access with a length of skin incision ranged from 37 to 52 cm. Hardware and autologous bone graft (local bone and iliac crest bone) was added in all the patients to stabilize the vertebral segments. Before surgery, we assigned an Infection Risk Index (IRI score) to each patient (Table 1). Every patient received antibiotic prophylaxis (1000 mg of cefazolin i.v.) 1 hour before surgery which was repeated every 3 hours during surgery, and the same dose 2 times/die for at least 48 hours was received until suction drainage removal. Before surgical incision, the skin was disinfected and prepared as usual, utilizing Poviderm solution, sterile drapes, sterile clothes, and gloves. Before surgery we randomly divided this group of 50 patients into two groups of 25 patients each: group A (11 males, 14 females; mean age 41.4 years) and group B (9 males, 16 females; mean age 42.1 years). In group A, before applying the bone graft, low-pressure irrigation (Bio Pulse, Leader Medica) with PVP-I diluted to a 3% concentration (30 g/l) in 2 litres of saline for between 5 and 10 minutes was performed and then washed out by 1 litre of sodium chloride solution through a pulse irrigation device. In group B, low-pressure irrigation with 2 litres of saline solution for between 5 and 10 minutes was performed before applying bone graft. Before and after pulsatile irrigation with diluted PVP-I in group A and before and after pulsatile irrigation with saline solution in group B, we obtained samples for bacterial cultures from muscular tissue in order to better evaluate the antimicrobial action of the solutions utilized in the 2 groups. The culture was made on blood agar plates for a sufficient number of days to allow for bacterial growth. Using the 2009 Centers for Disease Control and Prevention National Health Safety Network criteria [27], we clinically defined the presence of infected cases, and both superficial and deep SSIs were included. According to these criteria, superficial SSI occurred within 30 days after surgery and involves only the skin and the subcutaneous tissue of the incision, while deep SSI occurred within 30 days after surgery if no hardware is implanted or within 1 year if hardware is present and the infection seems to be related to operative procedure and involves deep soft tissue of the incision. SSI was detected if positive cultures and clinical signs of infection were found [27, 28].

The statistical analysis was not significant (*p* value = 0.235 by Fisher exact test).

## 3. Results

Groups A and B were homogenous regarding sex, age, and Infection Risk Index score. The duration of surgery above the 75th percentile (> of 239 minutes) (Table 2) was common to all patients. In group A, 19 had risk index score grade I and 6, grade II; in group B, 18 had risk index score grade I and 7, grade II. In group A, no infections were diagnosed despite the fact that the first samples obtained by swabs from muscular tissue, before performing intraoperative pulse irrigation, were found positive for wound contamination in 4 of the 25 patients (*Staphylococcus epidermidis* in 1,* Enterococcus faecalis* in 2, and* Escherichia coli* in 1). However, the microbiological cultures on the second samples obtained after the PVP-I and saline pulse irrigation did not confirm the contamination and did not show any bacterial growth (Table 3). In group B, we diagnosed 3 deep wound infections (6% of the entire cohort) (*p* < 0.05). In the infected wounds, we isolated* Escherichia coli* in 2 cases and* Staphylococcus aureus* in 1 case (Table 2). However in group B the samples before and after saline irrigation were positive in 4 cases* (2 Escherichia coli, 1 Staphylococcus aureus, and 1 Staphylococcus epidermidis)* (Table 3) but three of them (cases number 5, 6, and 8) developed SSI, while the case contaminated by* Staphylococcus epidermidis* (patient no. 1) did not develop clinical signs of wound infection in spite of previous wound contamination. At clinical presentation and after laboratory tests, all the infected patients were treated with surgical debridement, PVP-I and saline pulse irrigation, and at least 3 months of antibiotic therapy. The hardware was removed only in 1 of the infected patients, after the failure of the first surgical debridement (patient no. 6 in group B). At 2 years of follow-up after SSI treatment, we registered no signs of wound infection in any of these patients.

## 4. Discussion

Nowadays, postsurgical infections are becoming a serious problem also in developed countries as well for two main reasons: bacterium resistance to antibiotics and an increase in the number of surgical procedures. In the last 20 years, the number of spinal surgery procedures has increased and, although some of them are performed through minimally invasive technics, the risk of wound contamination and infection is still elevated and represents a very debilitating complication for the patient, with an increase of costs [29–33].

In a systematic review on the influence of perioperative risk factors and therapeutic interventions on infection rates after spine surgery, Schuster et al. [1] concluded that the causes of SSI are multifactorial and related to a complex interplay of patient and procedural influences.

As we found in our series of spinal operations, the risk was higher in patients with an IRI score > 0 (duration of surgery > 75% and ASA score > 2). In our patients, the ASA score was never more than 1, demonstrating a cohort in relative good general condition, while the index influencing infection risk was mostly the duration of surgery, always more than 4 hours (>75%). This correlation in increasing the risk of SSI was already underlined by Shiono et al. [34] with a study where they found that the probability of contamination by skin bacteria increases with the duration of surgery.

Povidone-iodine is an antiseptic agent with bactericidal activity against most pathogens including methicillin-resistant* Staphylococcus aureus* (MRSA) [35–38]. Cheng et al. [14] prospectively investigated 414 spinal surgery patients and compared wounds irrigated with PVP-I solution (3.5% concentration) to wounds irrigated with saline solution. They reported 0% infection rate in the PVP-I group and 3.4% of infection rate in the saline irrigated wounds (0.5% superficial infections and 2.9% deep infections).

Regarding cytotoxic effects of PVP-I, Kaysinger et al. [39] reported that the inhibitory effect of PVP-I on tibia and osteoblast cells isolated from embryonic chicks is significant only after exposure to concentrations of 5% betadine or higher. Recently Van Meurs et al. [40] in an in vitro study on antiseptic solutions for intraoperative irrigation reported that only diluted povidone-iodine was bactericidal at a concentration where some cell viability remained. They concluded that PVP-I diluted to a concentration of 1.3 g/l could be the optimal antiseptic for intraoperative irrigation.

In our study surgical wounds have been irrigated with PVP-I diluted to a 3% concentration (30 g/l) in normal saline solution, utilizing a pulsatile device. Experimental studies suggest that irrigation with high-pressure or low-pressure lavage may be effective for removing bacteria from contaminated wounds, but some studies have reported that high-pressure lavage can damage the bone and the surrounding soft tissues [26, 41–43]. In our patients, to reduce the risk of tissue damage, we opted for a low-pressure lavage in accordance with the result reported by Petrisor et al. [43], who examined “surgeons preferences in the initial management of open fracture wounds.”

The aim of our study was to prospectively evaluate the effect of wound irrigation with PVP-I and saline solution through a pulsatile device for the prevention of SSI after long-duration instrumented spinal surgery.

In group A samples obtained before pulse irrigation with Poviderm, we observed 4 cases of wound contamination, but no patient developed clinical signs of infection. On the other hand, when irrigation without PVP-I has been performed, wound contamination was present in 4 group B cases as well, but 3 (12%) of them developed SSI (*p* = 0.235).

However, contaminated surgical wounds do not always develop clinical signs of infection and patients with negative intraoperative cultures can develop postoperative infection [33, 34, 44]; in our series 3 wound infections have been diagnosed when PVP-I was not used and zero wound infections have been diagnosed when pulse irrigation with povidone-iodine has been performed.

## 5. Conclusions

In conclusion, it is clear that the causes of perioperative SSI are multifactorial, and they include specific patients and procedure risks. Because some of the risk factors are not modifiable, it is essential to maintain sterility during surgical procedure. Carefulness in wound sterility can be accomplished by the administration of pre- and intraoperative antibiotics and by the application of adjunct measures, such as PVP-I pulse irrigation. Although our cohort was homogeneous regarding general condition, mean age, surgical approach and technics, and duration of surgery, 8 cases were contaminated before wound closure; however, only 3 patients, not treated by PVP-I, developed SSI.

To the best of our knowledge, this is the first study that also uses bacterial cultures of samples obtained from the surgical site in order to show the efficacy of this adjunct method in preventing the risk of SSI.

This study can contribute to increasing the evidence of other available studies, by asserting that in order to reduce the risk of postoperative wound infection, pulse irrigation with dilute PVP-I and saline solution can be useful. However the number of the enrolled patients is small and a significant statistical analysis is not practicable; an additional study with a new group of patients is necessary to confirm these tantalizing preliminary results.

## Figures and Tables

**Table 1:** IRI score.

National Nosocomial Infections Surveillance
*ASA Score*
1-2: 0 points
3-4: 1 point
*Duration of surgery*
<75%: 0 points
>75%^**∗**^: 1 point
*Wound class*
I-II^**∗∗**^: 0 points
III-IV^**∗∗∗**^: 1 point

^**∗**^Above the threshold value of duration for that category of surgery (239 minutes); ^**∗∗**^clean, clean/contaminated; ^**∗∗∗**^contaminated, dirty/infected; IRI: Infection Risk Index; ASA score + duration of surgery + wound class = IRI score.

**(a):** 

Group A (PVP-I pulse irrigation)	Sex	Age	Spine pathology	ASA score	Wound class	Duration of surgery	IRI score	Wound infection
(1)	M	19	AIS	0	0	1	I	No
(2)	M	59	DS	0	0	1	I	No
(3)	F	40	AIS	0	0	1	I	No
(4)	M	30	AIS	0	0	1	I	No
(5)	F	68	DS	0	0	1	I	No
(6)	M	70	DS	0	0	1	I	No
(7)	F	39	AIS	0	0	1	I	No
(8)	F	50	AIS	0	0	1	I	No
(9)	F	70	DS	1	0	1	II	No
(10)	M	44	AIS	0	0	1	I	No
(11)	M	75	DS	1	0	1	II	No
(12)	F	61	AIS	0	0	1	I	No
(13)	F	20	AIS	0	0	1	I	No
(14)	F	66	DS	1	0	1	II	No
(15)	F	59	DS	1	0	1	II	No
(16)	M	14	IS	0	0	1	I	No
(17)	M	62	DS	1	0	1	II	No
(18)	F	14	IS	0	0	1	I	No
(19)	M	38	AIS	0	0	1	I	No
(20)	F	13	IS	0	0	1	I	No
(21)	F	13	CS	0	0	1	I	No
(22)	F	15	IS	0	0	1	I	No
(23)	M	27	AIS	0	0	1	I	No
(24)	F	16	IS	0	0	1	I	No
(25)	M	54	DS	0	0	1	II	No

**(b):** 

Group B (saline pulse irrigation)	Sex	Age	Spine pathology	ASA score	Wound class	Duration of surgery	IRI score	Wound infection
(1)	M	62	DS	0	0	1	I	No
(2)	F	51	AIS	0	0	1	I	No
(3)	M	46	DS	0	0	1	I	No
(4)	M	29	AIS	0	0	1	I	No
(5)	F	63	DS	1	0	1	II	Yes *E. coli*
(6)	F	20	AIS	0	0	1	I	Yes *E. coli*
(7)	F	21	AIS	0	0	1	I	No
(8)	F	71	DS	1	0	1	II	Yes *S. aureus*
(9)	M	60	DS	0	0	1	I	No
(10)	F	42	AIS	0	0	1	I	No
(11)	F	40	AIS	0	0	1	I	No
(12)	M	65	DS	0	0	1	I	No
(13)	F	73	DS	1	0	1	II	No
(14)	F	48	DS	1	0	1	II	No
(15)	F	50	DS	1	0	1	II	No
(16)	F	15	CS	0	0	1	I	No
(17)	M	27	AIS	0	0	1	I	No
(18)	M	41	AIS	0	0	1	I	No
(19)	F	66	DS	1	0	1	II	No
(20)	M	59	DS	1	0	1	II	No
(21)	F	17	IS	0	0	1	I	No
(22)	F	18	AIS	0	0	1	I	No
(23)	M	28	AIS	0	0	1	I	No
(24)	F	27	AIS	0	0	1	I	No
(25)	F	13	IS	0	0	1	I	No

AIS: adult idiopathic scoliosis, DS: degenerative scoliosis, IS: idiopathic scoliosis, and CS: congenital scoliosis.

**(a):** 

Group A (PVP-I pulse irrigation)	Infection index risk	Wound contamination (before pulse irrigation)	Wound contamination (after pulse irrigation)
(1)	I	Yes *(Staphylococcus epidermidis)*	No
(2)	I		
(3)	I		
(4)	I		
(5)	I		
(6)	I	Yes *(Enterococcus faecalis)*	No
(7)	I		
(8)	I	Yes *(Escherichia coli)*	No
(9)	II		
(10)	I		
(11)	II		
(12)	I		
(13)	I		
(14)	II	Yes *(Escherichia coli)*	No
(15)	II		
(16)	I		
(17)	II		
(18)	I		
(19)	I		
(20)	I		
(21)	I		
(22)	I		
(23)	I		
(24)	I		
(25)	II		

**(b):** 

Group B (saline irrigation)	Infection index risk	Wound contamination (before pulse irrigation)	Wound contamination (after pulse irrigation)
(1)	I	Yes *(Staphylococcus epidermidis)*	Yes *(Staphylococcus epidermidis)*
(2)	I		
(3)	I		
(4)	I		
(5)	II	Yes *(Escherichia coli)*	Yes *(Escherichia coli)*
(6)	I	Yes *(Escherichia coli)*	Yes *(Escherichia coli)*
(7)	I		
(8)	II	Yes *(Staphylococcus aureus)*	Yes *(Staphylococcus aureus)*
(9)	I		
(10)	I		
(11)	I		
(12)	I		
(13)	II		
(14)	II		
(15)	II		
(16)	I		
(17)	I		
(18)	I		
(19)	II		
(20)	II		
(21)	I		
(22)	I		
(23)	I		
(24)	I		
(25)	I		
